# Supplementary material for: A Pilot Cross-Sectional Study of Immunological and Microbiome Profiling Reveals Distinct Inflammatory Profiles for Smokers, Electronic Cigarette Users, and Never-Smokers
Source: Microorganisms. 2023 May 26;11(6):1405. doi: 10.3390/microorganisms11061405 (PMC10303504; doi:10.3390/microorganisms11061405)
Supplement: Supplementary file 1 [file microorganisms-11-01405-s001.zip › Manuscript 2 Supplemental Figures (updated April 2023).pdf]

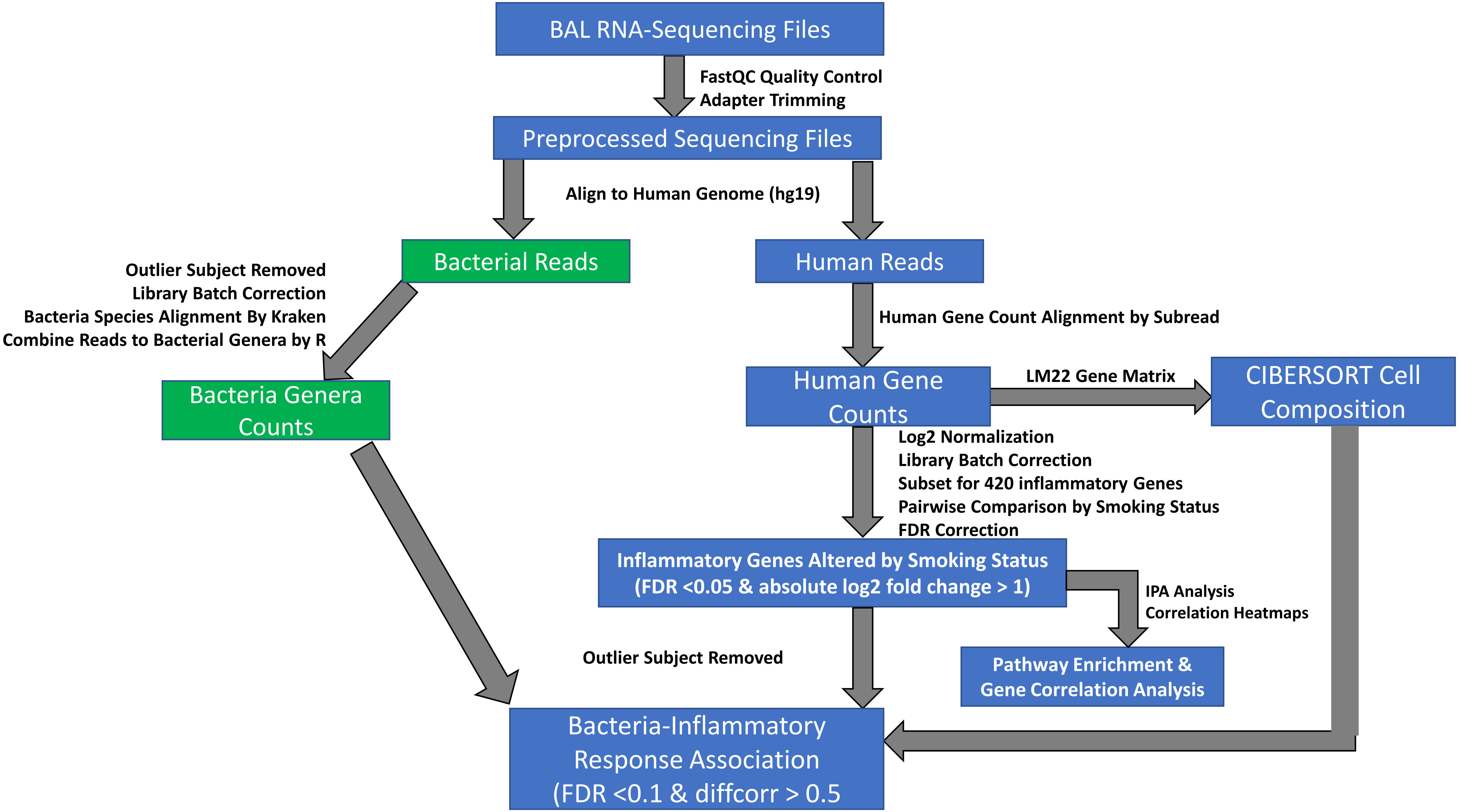

Figure S1. Diagram of study workflow. Study workflow by steps and details for human and bacteria analysis.

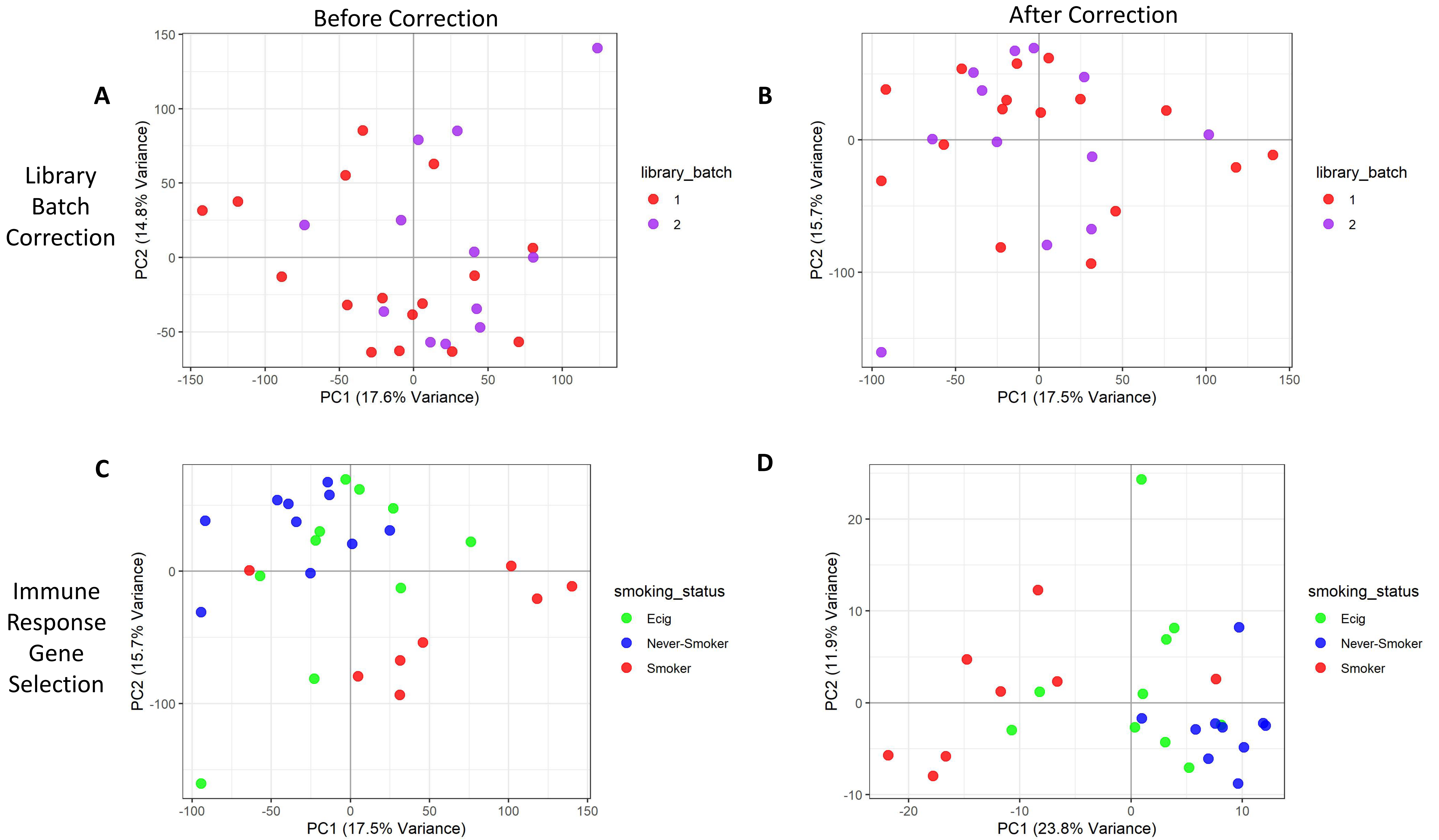

Figure S2. Principal component analysis (PCA) of human reads. A) PCA of human gene expression analysis before library generation correction. B) PCA of human gene expression analysis after library generation correction. C) PCA of 20517 human genes by smoking status after library generation correction. D) PCA of 420 immune genes by smoking status

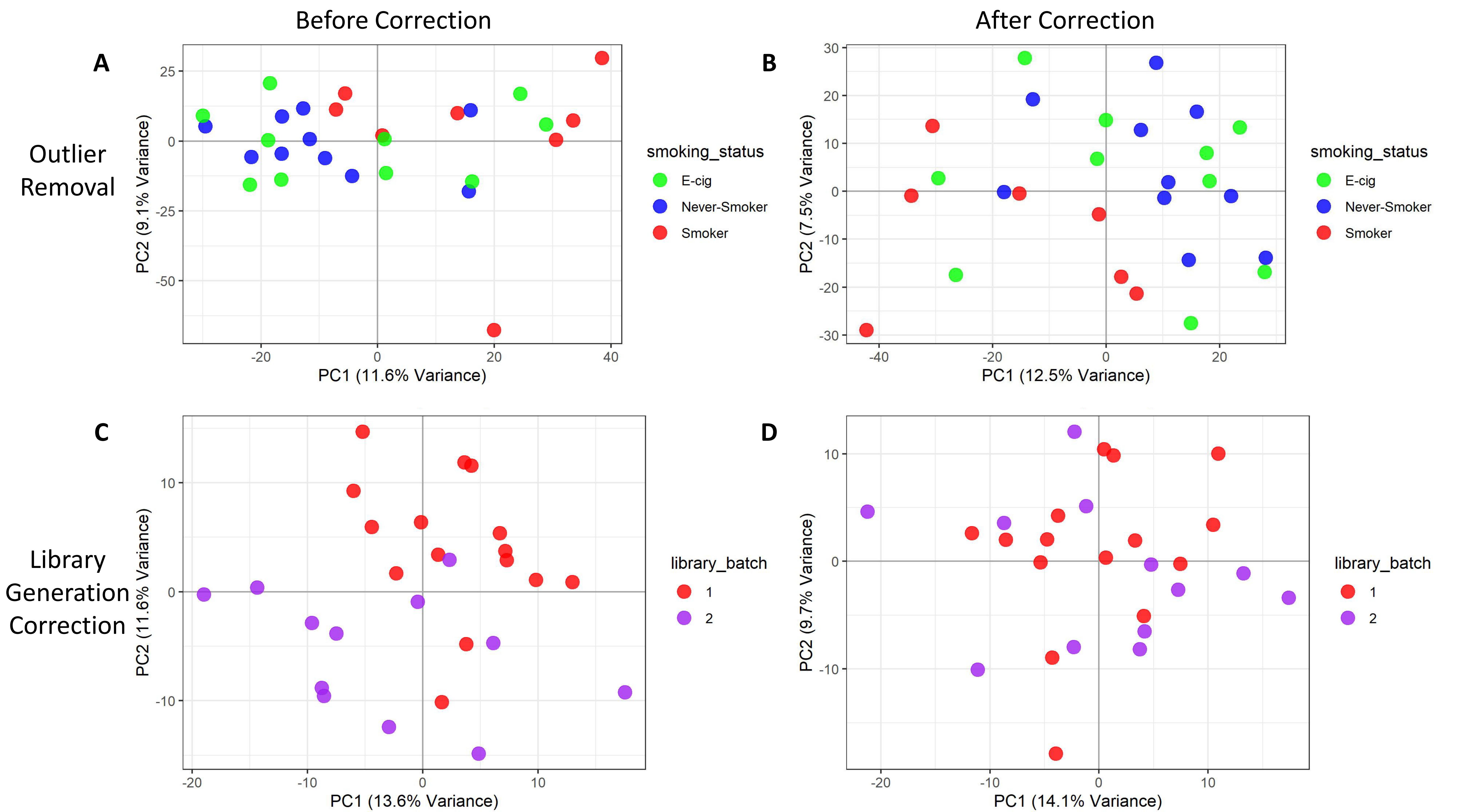

Figure S3. Principal component analysis (PCA) of bacteria genera analysis before and after outlier removal and library generation batch correction. A) PCA of bacteria genera analysis by smoking status before outlier removal. B) PCA of bacteria genera analysis by smoking status after outlier removal. After outlier was removed, library generation batch correction was observed. C) PCA of bacteria genera analysis before library generation correction. D) PCA of bacteria genera analysis after library generation correction.

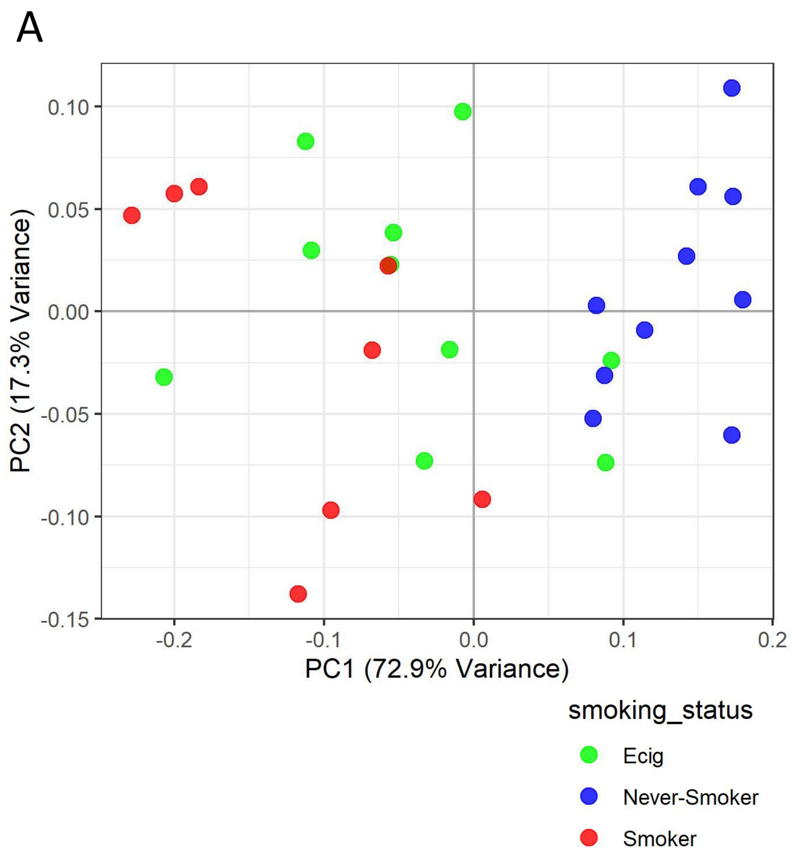

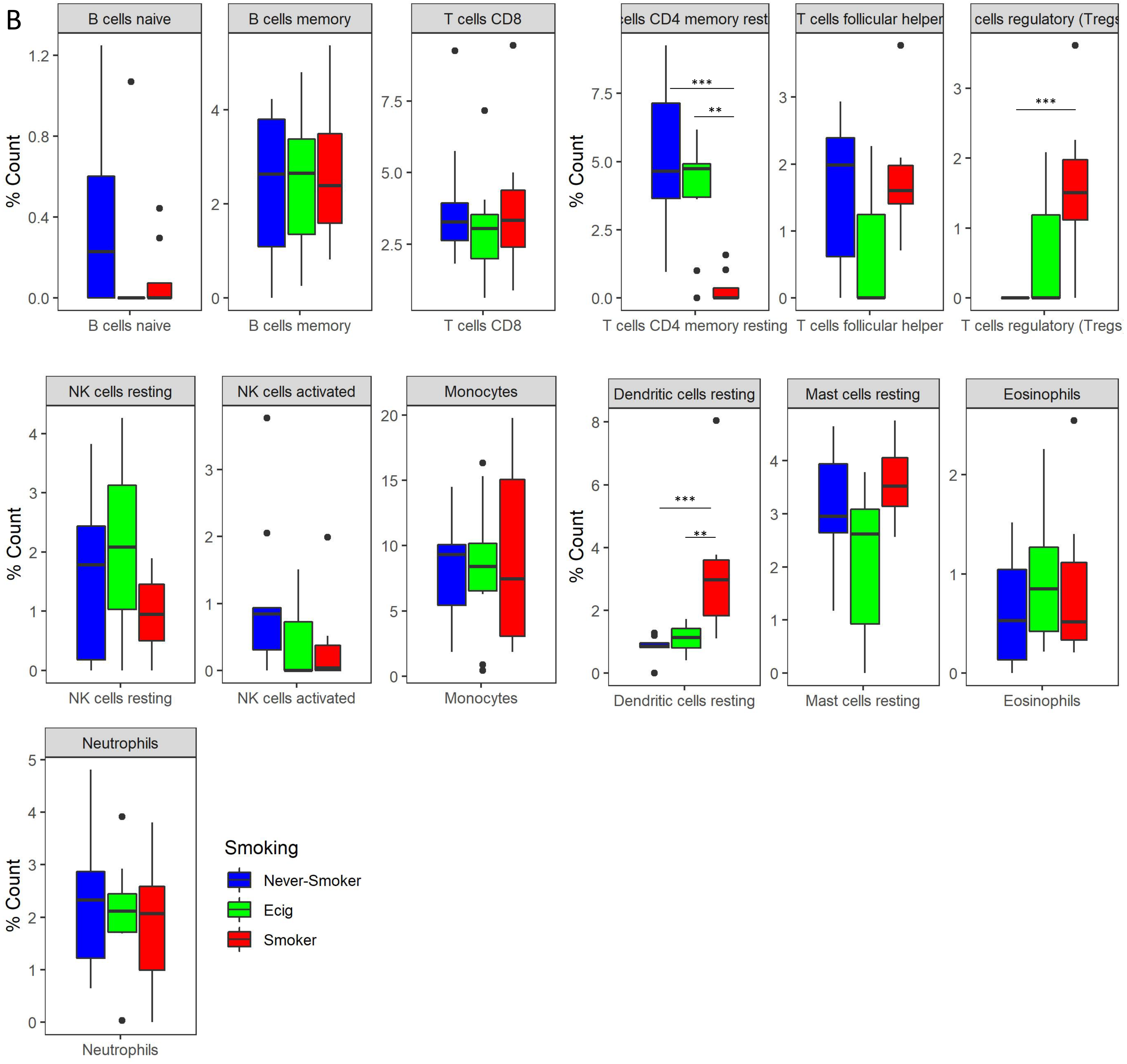

Figure S4. CIBERSORT Predicted Composition of Immune Cell Subtypes in BAL. A) PCA of overall cell composition as predicted by CIBERSORT B) Boxplots of predicted percent composition of 13 immune cell subtypes by smoking groups. Asterisks represent significance between pairwise smoking group comparison (**q-value < 0.01, ***q-value < 0.001, Dunn test). Due to at least 2 smoking groups having 0% cell composition, the following cell subtypes were not plotted: Plasma cells, T cells CD4 naïve, T cells CD4 memory activated, T cells gamma delta, Dendritic cells activated, and Mast cells activated.
